# Cross-Cultural Differences in Adopting Social Cognitive Career Theory at Student Employability in PLS-SEM: The Mediating Roles of Self-Efficacy and Deep Approach to Learning

**DOI:** 10.3389/fpsyg.2021.586839

**Published:** 2021-06-22

**Authors:** Wen-Xuan Zhao, Michael Yao-Ping Peng, Fang Liu

**Affiliations:** ^1^School of Economics and Management, Huaiyin Normal University, Huai'an, China; ^2^School of Economics and Management, Foshan University, Foshan, China; ^3^School of Foreign Languages, Huaiyin Normal University, Huai'an, China

**Keywords:** deep approach to learning, higher education, PLS-SEM, self-efficacy, student employability, teacher knowledge transfer

## Abstract

Unable to keep pace with the expectations of employers and societies that are constantly changing around the world, higher education policy and talent training have given rise to a gap between university education and employment. However, the higher education and industrial needs derived from technological progress have changed the development in country. This study aims to verify the learning method of Taiwanese vs. Malaysian university students and examines the relationship between teacher knowledge transfer and student employability from the perspective of a social cognitive career theory. In this study, 619 copies of questionnaires from the Taiwanese sample group and 443 copies of questionnaires from the Malaysian sample group were collected in total to compare the two sample groups in the development of student employability. The results indicate that teacher knowledge transfer has significant positive correlations with self-efficacy and a deep approach to learning and student employability, and the self-efficacy and a deep approach to learning have significant positive correlations with student employability in the Taiwanese sample. In Malaysia, except for the path between teacher knowledge transfer and student employability, all paths were significant and positively related. Finally, according to our results and findings, this study proposes several insights with practical and theoretical implications for future study.

## Introduction

Scholars in multiple disciplines have been focusing on the concept of student employability. Studies have confirmed the importance of the development of student employability (Sin et al., 2019). De Vos et al. (2011) suggested that student employability is acquired by students through developing skills, knowledge, and capacity to meet the talent demands of the employment market. For individuals, this ability to work is essential because working provides purpose in life, financial security, and social contacts (Schuring et al., 2011). Donald et al. (2019) indicated that definitions proposed by different scholars may vary from each other because of differences in research contexts. For example, Rothwell and Arnold (2007, p. 25) defined employability as the individuals' ability to maintain the job one person has, or to get the job one person desires, whereas Vanhercke et al. (2014, pp. 594, 599) defined perceived employability as the individual's perception of his/her possibilities of obtaining and maintaining employment. According to the views of Donald et al. (2019) and Tavares (2017), this study aims to explore the undergraduate self-perception of employability. However, population aging (Fisher et al., 2017) and rapid changes in technology and changes in the nature of work (Van der Klink et al., 2016) complicatedly promote student employability of the working population during extended working lives (van Dam et al., 2017). Therefore, a thorough scientific understanding of student employability and its determinants is necessary (Donald et al., 2019).

Indeed, universities must improve student employability through factors of operation that remain to be clarified, but it is important to note that student employment rates can increase the visibility of universities, which is a focus for these universities (Pan and Lee, 2011; Ahmed et al., 2015; Cacciolatti et al., 2017; Blázquez et al., 2018; Sin et al., 2019). However, there are few studies that have examined the development of student employability from a wide range of cross-cultural perspectives (Sheu et al., 2014; Presti et al., 2018). As cultural differences are the main obstacles for higher education institutions (HEIs) to develop internationally accepted skills and capabilities in student learning (Sin et al., 2019), understanding the differences also facilitates specific higher educational strategies for students (Sheu et al., 2014; Presti et al., 2018). This study aims to explore this issue with the intention to help HEIs in two countries—Taiwan and Malaysia—understand the antecedents affecting the development of student employability.

According to Bandura (1997) and social cognitive theory (SCT), personal attributes, environmental influences, and intentional behaviors form a triangular relationship (Cupani et al., 2010), wherein individual behavior is formed *via* the interactions among individual thoughts and environmental emotions (Peng et al., 2018). Built on the foundation of SCT, social cognitive career theory (SCCT) was proposed to explain the development between influencing factors and satisfaction in higher education (Lent et al., 1994; Lent and Brown, 2006; Burga et al., 2020). In the SCCT, environmental and behavioral factors have indirect effects on personal and cognitive factors. In regard to environmental factors (Lent et al., 2011; Duffy et al., 2014; Chang and Edwards, 2015; Thompson et al., 2017; Liguori et al., 2019), several previous studies have suggested that the skills development, knowledge acquisition, and capacity construction of university students are mostly related to the “teacher factor” (Peng et al., 2018). That is, students need teachers who can provide courses and curricula based on employment trends, ethical values, career planning, work features, and even student employability to reduce the academia–employment gap (Cacciolatti et al., 2017). Teacher's teaching and student's learning are interactive processes, which are established on the basis of good interaction. Students are able to gain important knowledge from teacher's teaching process (Sin et al., 2019) and internalize this knowledge, and the concept is similar to knowledge transfer (Astorga-Vargas et al., 2017). Moreover, previous literature on knowledge transfer is oriented by knowledge provider and knowledge acquisition in terms of concept statements (Guldberg et al., 2017; Steins and Behravan, 2017), emphasizing the interaction between knowledge provider and knowledge gainer. Thus, teachers play a vital role in the process of student learning. Teachers' teaching styles and modes are also some of the determining factors for whether students can obtain knowledge and capabilities from the learning process (Guldberg et al., 2017; Steins and Behravan, 2017). This implies that the contents of knowledge transferred from teacher vary the differences of development of student employability. Thus, this study aims to explore the antecedent role of teacher knowledge transfer in a research framework of SCCT.

In addition, in the SCCT, self-efficacy is a key factor in inspiring spontaneous learning involvement and engagement (Caesens and Stinglhamber, 2014), as well as the core of the SCCT (Lent et al., 2011; Sheu and Bordon, 2017; Thompson et al., 2017; Jemini-Gashi et al., 2019; Liguori et al., 2019). Self-efficacy refers to the students' belief in their successful performance and education-related behaviors and capabilities that are related to the initiation of spontaneous learning engagement and motivation (Komarraju and Nadler, 2013; Bocanegra et al., 2016). Except for self-efficacy, the level of students' learning engagement is focused on a learning process of transforming knowledge into skills and capabilities. Smart et al. (2000) regarded learning engagement as a mediator that can connect academic development with learning outcomes. To understand learning engagement more specifically, Marton and Säljö (1976) proposed a deep process that can effectively connect learning engagement to learning content (Dolmans et al., 2016). National Research Council (NRC) (2012) defines deep approach to learning as the process through which a person becomes capable of taking what was learned in one situation and applying it to new situations. Deep approach to learning can help students to explore knowledge beyond the information itself (Lindblom-Ylänne et al., 2019), enabling students to engage in an effective learning environment (Varunki et al., 2017). Students with a greater deep approach to learning are able to process, integrate, and absorb useful knowledge more quickly (Dolmans et al., 2016; Varunki et al., 2017), and student employability will be enhanced accordingly when students have a higher efficiency and efficacy of using knowledge. Therefore, this study suggests that combining the teacher knowledge transfer, self-efficacy, deep approach to learning, and SE concepts of SCCT can help to address the deficiencies of prior literature.

Differences in national cultures bring about different values, norms, and beliefs, which are put forward by national citizens in different cultures, and may result in huge differences in students' learning and psychological status. In order to explore the teaching and learning activities caused by differences of culture and changes of student employability, students in Malaysia and Taiwan were taken as the research samples of the interregional comparison to learn about the relevance of the research variables. Therefore, this study focuses on determining university students' perceptions of the drivers of employability, self-efficacy, and deep approach to learning in higher education, as well as the relationships among them.

## Literature Review

### Theoretical Background of Social Cognitive Career Theory

The SCCT is the initial foundation in this study to evaluate the effective teacher knowledge transfer toward students' competence enhancement (Lent et al., 2011; Chang and Edwards, 2015; Jemini-Gashi et al., 2019; Liguori et al., 2019). The SCCT is an empirically validated model that has been widely accepted (Brown et al., 2011; Duffy et al., 2014; Burga et al., 2020). It is a method for understanding and predicting changes in human behaviors and cognitive behaviors (Bocanegra et al., 2016). According to this theory, human metadevelopment occurs through continuous interaction with the external environment, and the environment must go through a cognitive process before affecting human behaviors (Lent et al., 2011; Duffy et al., 2014; Chang and Edwards, 2015; Thompson et al., 2017; Liguori et al., 2019).

According to Lent et al. (1994), self-efficacy is the key structure of SCCT and is believed to have a direct impact on behavior (Brown et al., 2011; Duffy et al., 2014; Chang and Edwards, 2015; Liguori et al., 2019). The outcome expectation is the second structure of SCCT, representing a person's judgment on the consequences resulting from the execution or non-execution of a specific behavior (Brown et al., 2011; Caesens and Stinglhamber, 2014; Duffy et al., 2014). The pattern of manifestation of outcome expectation can be embodied as self-perception such as student employability (Thompson et al., 2017). The goal is the third core structure of SCCT and can have a direct impact on behavior and regulate other structures in the model. Achievement of goals requires specific self-regulation skills, such as gaining employability and completing specific goals (Brown et al., 2011; Lent et al., 2011; Caesens and Stinglhamber, 2014; Duffy et al., 2014).

Although Lent et al. (1994) clearly described a social cognitive career structural network self-efficacy in the past studies that has received more attention than other model groups or examined by only one or two other variables (Brown et al., 2011; Duffy et al., 2014; Chang and Edwards, 2015). This study believes that self-efficacy cannot be studied in isolation (Lent et al., 2011; Caesens and Stinglhamber, 2014; Jemini-Gashi et al., 2019; Liguori et al., 2019). We will use SCCT framework to further understand the impact of teacher knowledge transfer on student employability among Taiwanese and Malaysian students (Hansen et al., 2012; Chang and Edwards, 2015; Thompson et al., 2017). More specifically, the purpose of this study is to examine the impact of teacher knowledge transfer on self-efficacy, deep approach to learning, and student employability; analyze its relationship with student employability; and determine whether the effect arising from such a relationship varies with cross-cultural perspectives (Sheu and Bordon, 2017; Presti et al., 2018).

### Student Employability

Student employability has attracted increasing academic attention in recent years. Employability refers to an individual's capability to be employed in work. Although quite basic, this definition does match scientific definitions of employability (Hennemann and Liefner, 2010). For example, Fugate et al. (2004) refer to employability as “one's ability to identify and realize career opportunities” (p. 23). What these and more historical definitions of employability have in common (Makkonen, 2017) is that employability is an individual characteristic that is determined by various (internal and external) factors (Fugate et al., 2004; Vermeulen et al., 2018; Shahzad et al., 2020) and describes how well an individual is capable of becoming employable and maintaining employability. As such, employability should capture an individual's capacity to function in the field of higher education (Ahmed et al., 2015; Cacciolatti et al., 2017; Blázquez et al., 2018). Based on above arguments, student employability can be defined as the students' appropriate application of competence, continuous acquisition and creation of essential work skills in order to accomplish all the tasks, and adaptation to labor market changes.

While simple, the aforementioned definitions capture the role of time but also potentially added value for employers as well (Hennemann and Liefner, 2010). First, the inherently longitudinal component of student employability is explicated in the aforementioned basic definition of student employability by considering an individual's employability over time. As what will be discussed later, the existing conceptualizations of student employability have not yet explained this aspect to the same extent. Student employability is conceptualized in a sufficiently broad way; this is required by the longitudinal aspect of student employability, as knowledge and learning are crucial for an individual's long-term ability to function in the learning process and in university (Cacciolatti et al., 2017). As employers and society are the main stakeholders, the focus of employability is also relatively clear. That is, students with higher employability are beneficial to employers, and the society benefits from high employment rates. In measurement, student employability can be regarded as a higher-order construct (Pan and Lee, 2011). Pan and Lee (2011) suggested a measurement of employability that includes general and professional abilities required at work: work attitude, career planning ability, and confidence.

### Teacher Knowledge Transfer

According to the cognitive learning perspective, students can leverage their knowledge and resources to build their own capabilities and shape their employability through the use of their abilities. Teacher knowledge transfer can effectively facilitate students' knowledge to be applied in the management process to create value (Walter et al., 2007; Guldberg et al., 2017; Steins and Behravan, 2017). Therefore, in the learning process, students must internalize the information. The human mind is where knowledge comes into being by means of learning or experience and has gradual growth along with experience, such as personal beliefs, judgments, and value perceptions, in addition to explicit textual behavior, which includes the implicit mental journey (Steins and Behravan, 2017). Polanyi (1962) made a distinction between explicit knowledge (EK) and tacit knowledge (TK). There are a variety of physical and electronic formats for EK to be encoded and stored that are objective and rational, whereas TK is hard to be presented and represents one's own experience, reflections, cognitions, or talents (Astorga-Vargas et al., 2017).

In regard to knowledge transfer, scholars have suggested four steps: (1) socialization: the process of TK, the convenience of life experiences, and the capacity where students reside and are needed for the beginning of knowledge transfer; (2) externalization: from tacit to explicit, the knowledge is changed, making the activities in groups, which are conducted for the facilitation of knowledge management, to be propitiated (Nonaka and Von Krogh, 2009; Zhou et al., 2010); (3) combination: a process where new EK is created and merged from diversified pieces of current EK; and (4) internalization: a process in which something learned from EK is dedicated to practical use by the students (Astorga-Vargas et al., 2017). In the learning process, internalization represents how students apply TK and EK in real life in order to solve practical problems. It can be known from the above four stages that the contents of knowledge transfer focus on two important knowledge sources: TK and EK, which are also two significant factors for teacher knowledge transfer. Therefore, based on the above statements, teacher knowledge transfer can be considered as a learning process. Teachers will transfer TK and EK to students through various teaching modes, enabling students to integrate it with their own currently held knowledge.

Teacher knowledge transfer facilitates students to acquire more knowledge. As mentioned previously, teacher knowledge transfer is regarded as a learning process that includes changes in the learning environment, course assignments, and the conversion of teacher's instruction style (Guldberg et al., 2017; Steins and Behravan, 2017). According to the characteristics of teacher knowledge transfer, while TK is more ambiguous than EK, teachers use learning patterns to assist students in acquiring the value of knowledge (Cacciolatti et al., 2017). In short, the utilization of EK helps to enhance general working abilities and professional working abilities while promoting learning efficiency (Steins and Behravan, 2017), thereby enhancing students' academic attitudes and confidence to achieve course tasks. EK plays an indispensable role in general and professional competence (Fugate et al., 2004), but the most important aspect of this is its combination with TK to increase creativity (Blázquez et al., 2018). Moreover, Teigland and Wasko (2009) claimed that teacher knowledge transfer can help students to reuse knowledge, solve general problems, interact with teachers, and create new knowledge; the transformation and combination of TK can also generate new and novel ideas. In other words, more content in teacher knowledge transfer helps students to acquire the knowledge and know-how that are used to build their own employability (Cacciolatti et al., 2017). Thus, this study proposes the following hypothesis:

*H1: Teacher knowledge transfer has a positive and significant impact on student employability*.

According to the SCCT, information that comes from enactive mastery of experiences, vicarious (observational) experiences, social persuasions, and the status of physiology and psychology allows for students' self-efficacy creation (Van Dinther et al., 2011). Transferred from the teacher, experience mastery offers students with authentic evidence that convinces them that they can complete the task and therefore plays a significant role in strong self-efficacy creation (Van Dinther et al., 2011). Students interpret and identify their activities, and based on that, they develop the capacity for future tasks (Guldberg et al., 2017). Transferred from the teacher, TK and EK is delivered to students and provides affirmative persuasion that they can achieve the task. Efficacy generation and adherence are more accessible, particularly in the case of knowledge-specific tasks, if teachers deliver more significant knowledge into students' capacities and the enhancement of confidence. Based on the above arguments, this study proposes following hypothesis:

*H2: Teacher knowledge transfer has a positive and significant impact on self-efficacy*.

With respect to the formation of cognition, identity, and behavior, teacher knowledge transfer is effective in being recognized in a wide range of studies concerning with psychology and higher education, as well as in emphasizing learning involvements (White et al., 2008). While doing educational research, much more comprehension of abundant learned knowledge has been established by scholars. To complete a specific task, particularly on the basis of observational study on the behaviors of teacher knowledge transfer, a base of more effective learning methods is needed for students (Oleson and Hora, 2014; Guldberg et al., 2017), such as a deep approach to learning. These TK and EK learning activities may be shaped by the influence of teachers (Steins and Behravan, 2017), learning approaches, and experiential knowledge in a course (Pike et al., 2012). This implies that students are more capable of further thinking over how to apply the acquired knowledge and how to integrate attributes of different knowledge and rethinking knowledge connotation and difference when students have a rich and valuable basis of TK and EK. In other words, good teacher knowledge transfer is conducive to enlightening students to learn with higher initiative and enthusiasm and systematically integrate and rethink knowledge contents. As mentioned previously, teacher knowledge transfer is a learning process that includes changes in the learning environment, assignment of course tasks, or conversion of the teacher's instruction style. In particular, these learning approaches and belief systems are impressed in the students' minds while they receive wanted knowledge from teachers (Dolmans et al., 2016). In the initial learning phase, students continually acquire, integrate, and reflect on the specific knowledge delivered from instructors as apprentices; they further logistically conduct imitation. Conversely, episodic recall makes a set of acceptable behavioral scripts available. This knowledge learning process implies that students conduct a series of deep learning behaviors in relation to their achievement (Dolmans et al., 2016), engagement, and learning. On this basis, this study proposes the following hypothesis:

*H3: Teacher knowledge transfer has a positive and significant impact on deep approach to learning*.

### Self-Efficacy

Social cognition scholars argue that individuals' behavioral outcomes will be influenced by both environmental and cognitive factors in a given situation (Van Dinther et al., 2011), especially those beliefs that lead to success and behavior (Lent et al., 2014; Wang et al., 2016). They regard these beliefs as self-efficacy, an important cognitive variable in the process of interpreting individual formative behaviors and interacting with the environment (Komarraju and Nadler, 2013; Lent et al., 2014; Sheu et al., 2014). It can also be seen as the basis for human behavioral motivation, mental health, and personal achievement (Dacre Pool and Qualter, 2013; Burga et al., 2020). Self-efficacy is widely used in the field of education to explore the psychological cognitive factors of students of different ages and their positive impact on academic achievement and student career development (Komarraju and Nadler, 2013; Wang et al., 2016; Burga et al., 2020).

According to the above discussion, students who have confidence in their abilities will have more efficient behavior and better interpersonal relationships than those who do not. According to Dacre Pool and Qualter (2013), students with high self-efficacy look for resources and opportunities to accomplish tasks and further facilitate the development of related capabilities and skills (Fugate et al., 2004; Van Dinther et al., 2011; Komarraju and Nadler, 2013; Burga et al., 2020). Only by establishing and maintaining self-efficacy can they achieve their goals through leverage of knowledge and resources (Lent et al., 2014; Sheu et al., 2014). Furthermore, self-efficacy can also be seen as a strong and positive self-cognition and the process of solving problems and achieving tasks for students through high self-efficacy that positively affect their employability (Cacciolatti et al., 2017; Burga et al., 2020). According to the above, this study proposes the following H4:

*H4: Self-efficacy has a positive and significant impact on student employability*.

Some scholars have focused their investigations on mental health concerns, learning engagement, and involvement in college students (Song and Ingram, 2002; Tong and Song, 2004). However, few studies thus far have evaluated this population's general self-efficacy and learning engagement (Evans et al., 2017). Previous studies indicated that learning modes engaged by students can be divided into several groups, such as surface learning, deep learning, exploitative learning, or explorative learning (Dolmans et al., 2016; Varunki et al., 2017). According to different situations, different learning modes have varied corresponding effects. Some scholars suggest that students with high learning engagement tend to have high levels of effort expectancy because they are more interested in new knowledge and are more willing to learn how to integrate, combine, and absorb knowledge through deep approach to learning (Varunki et al., 2017). To facilitate students to engage in deep approach to learning, it is necessary to strengthen self-confidence in achieving learning goals and tasks (Komarraju and Nadler, 2013; Dolmans et al., 2016; Burga et al., 2020). Scholars suggest that the key for students in using deep approach to learning for effective learning is the need for a high degree of psychological and cognitive support, so that they can consciously engage in knowledge integration, reflective learning (RL), and problem-solving related to learning activities. In other words, students with higher self-efficacy have more intention to engage in deep approach to learning (Komarraju and Nadler, 2013). Therefore, the study proposes the following hypothesis:

*H5: Self-efficacy has a positive and significant impact on students' deep approach to learning*.

### Deep Approach to Learning

Deep approach to learning, a term used to describe students' learning efficiency, was initially put forward by Marton and Säljö (1976). Biggs (2003) described the behavioral characteristics of deep learning and surface learning (Dinsmore and Alexander, 2012; Dolmans et al., 2016): Deep learning aims to understand, explain, critically evaluate, connect, and integrate one concept with another, whereas surface learning usually adopts memory and rehearsal strategies (Laird et al., 2006, 2008; Dolmans et al., 2016; Varunki et al., 2017). In contrast, deep approach to learning is to develop the deep and concrete teaching mode through the cooperation of students, colleges, universities, and instructors (Lindblom-Ylänne et al., 2019), such as inducing positive student responses, building student prior knowledge, and teaching more ideas and interconnectedness between ideas (Biggs and Tang, 2011; Campbell and Cabrera, 2014; Lindblom-Ylänne et al., 2019). Since then, studies on deep approach to learning have been increasing, and different researchers have explained the concept of deep approach to learning from diverse perspectives. In the report by the National Research Council (2012), the following methods were proposed to promote deep learning: (1) use multiple and varied representations of concepts and tasks; (2) encourage elaboration, questioning, and explanation; (3) engage learners in challenging tasks; (4) teach with examples and cases; (5) prime student motivation; and (6) use “formative” assessments. The National Research Council (NRC) (2012) divided learners' development abilities in deep learning into the cognitive domain (including thinking, reasoning, and related skills), the intrapersonal domain (including the ability to regulate one's behavior and emotions to reach goals), and the interpersonal domain (involves expressing information to others, as well as interpreting others' messages and responding appropriately). Recently, deep approach to learning has received the attention of scholars and HEIs and has been used to inspire students' potential (Tagg, 2003; Biggs and Tang, 2011; Campbell and Cabrera, 2014; Varunki et al., 2017). In deep approach to learning, students not only pay attention to the basic content but also emphasize the basic meaning of information, mutual connection, knowledge integration, and metacognition (Biggs and Tang, 2011; Pascarella et al., 2013; Varunki et al., 2017). Therefore, the nature of deep approach to learning is to integrate and aggregate previously learned information and turn it into a part of personal thinking (Lindblom-Ylänne et al., 2019), thereby examining new phenomena and activities from different perspectives and perspectives.

While reviewing studies of deep learning, Dinsmore and Alexander (2012) found that 48% of the studies used a questionnaire survey to determine whether deep learning occurs and the degree of deep learning, including the Study Process Questionnaire (SPQ), the Learning Process Questionnaire, the Inventory of Learning Process, and the Approaches and Study Skill Inventory for Students. Most of these questionnaires were focused on the learning process to evaluate whether students used the deep learning method. Campbell and Cabrera (2014), Biggs and Tang (2011), and Pascarella et al. (2013) adopted the SPQ from Biggs (2003) and deconstructed deep learning into three dimensions of higher-order learning (HOL), integrative learning (IL), and RL to create a questionnaire. What the HOL has underlined is that students consider progressive skills in thinking as something brought by their curriculum. Students engage themselves in activities in abundant circles, constituting IL, which leads to the integration of ideas and various opinions from different sources. RL refers to the learning and expansion of students' comprehension and understanding based on their own ideas, as well as the consequent application of their new knowledge in life (Laird et al., 2008; Pascarella et al., 2013). This study adopts the measurement proposed by Biggs and Tang (2011), Campbell and Cabrera (2014), and Pascarella et al. (2013), dividing deep approach to learning into HOL, IL, and RL.

This study shows that student employability achieved by deep approach to learning presents a significant improvement. Previous literature has focused on the comparison between deep and surface learning; there has been a lack of studies on whether deep approach to learning can effectively improve student employability (Cacciolatti et al., 2017; Varunki et al., 2017). However, research results showed that deep approach to learning was positively related to student learning outcomes, knowledge integration, learning engagement, and skills (Laird et al., 2006, 2008). This also reflects that in the HEIs with a well-established learning assessment (Dolmans et al., 2016), students tend to use deep approach to learning to obtain more EK. A series of general knowledge of learning processes, HOL, IL, and RL can help students face difficult circumstances, deal with course tasks, understand new knowledge, and further improve core competence (Oleson and Hora, 2014; Dolmans et al., 2016; Varunki et al., 2017). In addition, deep approach to learning guides students to pay attention to both knowledge acquisition and the acceleration of substantial learning and comprehension of its fundamental significance (Laird et al., 2006, 2008; Cacciolatti et al., 2017). This all contributes to enhancing the critical thinking of students, problem-solving, and other skills related to employment (Pascarella et al., 2013). Therefore, the study proposes the following hypothesis:

*H6: Deep approach to learning has a positive and significant impact on student employability*.

**Figure 1:**
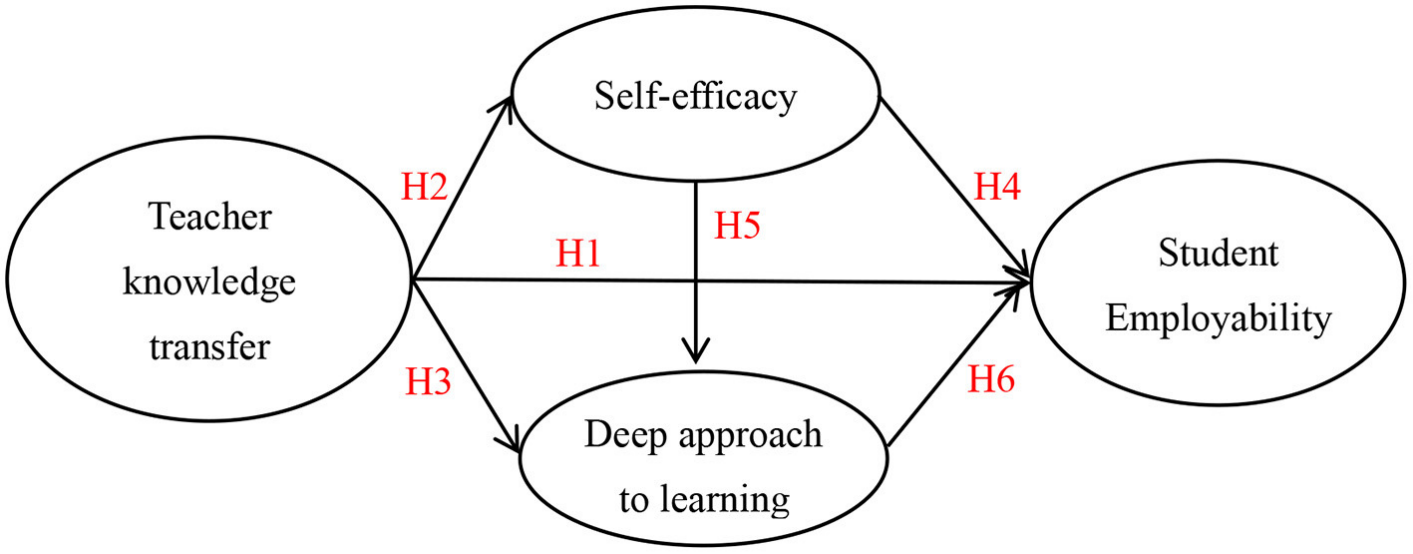
Research framework.

The research framework is shown in Figure 1.

## Methodology

### Participants and Sampling

This study is involved in conducting a questionnaire survey of university students in Taiwan and Malaysia. In cross-cultural comparison, there are two reasons for taking Taiwan and Malaysia as the sample sources. First, despite that there are cultural differences between Malaysia and Taiwan, the sample objects are mainly ethnic Chinese, who have a small language gap, and can understand items in the same questionnaire. Second, there are similarities for Malaysia and Taiwan in terms of higher educational forms, such as curriculum design and teaching patterns. It is of research value to explore the influence of cross-cultural differences on the learning process. Because of the large number of HEIs in Taiwan and Malaysia, it is difficult to perform tests on all of the HEIs in both countries; therefore, purposive sampling was employed. In addition, to accurately measure university students' perceptions of the variables in the study and to enhance the study's external validity, some principles for sampling were set. First, junior and senior students who had adapted to university life were taken as respondents, as freshmen and sophomores may not be able to clearly express their employment intentions, making it impossible to measure the effect of each variable on student employability (Peng, 2019). Second, considering that the sample needed to comprise students with clear employment orientations, the question, “Do you intend to pursue further study?” was included in order to exclude students who were less likely to seek work in the near future, enhancing the representativeness of the sample.

Using telephone and email, the researchers initially contacted universities and teachers to inquire if they were willing to ask their students to complete the questionnaire. Before completing the questionnaires, students were asked if they understood their rights regarding the survey, in order to meet ethical requirements. This study selected 10 Taiwanese HEIs and 10 Malaysian HEIs and then sent 2,000 copies of questionnaire to each of them. The students voluntarily completed the questionnaire, after signing their informed consent. During the school year (September 2018–January 2019), students completed the questionnaire. After sampling, 619 copies of questionnaire from Taiwanese and 443 copies of questionnaire from Malaysian were returned in total, with an effective response rate of 61.9 and 44.3%. The study focused on respondents from the social sciences (62.3% in total, in which Liberal Arts accounts for 7.6%, Management accounts for 31.4%, and Education accounts for 23.3%) and the natural sciences (38.7% in total, in which Science accounts for 7.2%, Engineering and Computer Science account for 17.6%, Life Science accounts for 7.3%, Bioresource and Agriculture account for 3.1%, and Technology accounts for 3.5%). This simplified the analysis process and kept the research focused. Before the cross-cultural comparative analysis, this study first verifies whether different background variables lead to differences in Taiwanese or Malaysian samples or not. Because of the different types of HEIs and disciplines, a systematic error might have arisen, bringing the study's external validity into question. Thus, several independent-samples *t*-tests were used to verify whether the groups of social sciences vs. natural sciences differed significantly in terms of the research dimensions. The results indicated that the groups did not significantly differ, so it was deemed appropriate to merge the samples from different universities and disciplines in each Taiwanese and Malaysian sample. Table 1 shows descriptive statistics of Taiwanese and Malaysia samples.

**Table 1:** Descriptive statistics of Taiwanese and Malaysia samples.

**Characteristic**	**Scale**	**Taiwan**	**Malaysia**
Gender	Male	310	224
	Female	309	219
Part-time job	Yes	389	342
	No	230	101
Scholarship	Yes	272	215
	No	347	228
First-generation college student	Yes	412	282
	No	206	161
Majors	Social science	347	230
	Natural science	272	213
Dedication to class preparation	Yes	336	132
	No	283	211
Weekly study hours spent on major courses	<5	401	142
	5 to <10	103	123
	10 to <15	42	85
	15 to <20	38	72
	>20	35	21

This study hid the names of constructs and assigned the question items randomly to prevent common method variance (CMV). The Harman one-factor analysis method was used to test for CMV. The explained variance in one factor was 33.72%, which is smaller than the recommended threshold of 50%. Therefore, CMV was not problematic in this study (Podsakoff and Organ, 1986).

### Instrument

Student employability is a higher-order construct that includes subjective and objective aspects (Fugate et al., 2004; De Vos et al., 2011; Pan and Lee, 2011). In the objective aspect, the Department of Education (Department of Education, 2006) established an “employability skills framework” with eight categories: communication skills, teamwork ability, problem-solving ability, original and entrepreneurial ability, planning and organizational ability, self-management ability, autonomous learning, and scientific and technological ability. In the subjective aspect, some scholars have developed measurement scales to examine individual cognition on employability in several ways (Andrews and Higson, 2008; Pan and Lee, 2011). This study included the general ability for work (eight items), professional ability for work (PAW) (four items), attitude to work (AW) (three items), and career planning and confidence (three items) measures, as proposed by Pan and Lee (2011).

Teacher knowledge transfer measurement items were revised proposed by Zhou et al. (2010). The wording of this scale was modified from that of the study from Zhou et al. (2010) to fit the classroom setting. Thus, it included EK (five items) and TK (four items).

Self-efficacy is an individual's perception that they will achieve a goal before starting the necessary tasks. Self-efficacy has a considerable influence on the choice of tasks, level of task performance, effort made to finish tasks, and persistence regarding task performance. The scale developed by Rigotti et al. (2008) was revised to integrate six items of higher reliability and validity.

For deep approach to learning, the scales developed by Campbell and Cabrera (2014), Laird et al. (2006, 2008), and Pascarella et al. (2013) on the basis of national survey of student engagement (NSSE) items were adopted: HOL (four items), IL (five items), and RL (two items). All items were measured on a five-point Likert scale (1 = totally disagree, 5 = totally agree) and are shown in Table 2.

**Table 2:** Instruments description.

**Construct**	**Variables**	**Items**
Student employability	General ability for work	Expression and communication
		Time management
		Leadership
		Innovation
		Team work
		Native language
		Foreign language
		Stability and pressure resistance
	Professional ability for work	Professional knowledge and skill
		Computer literacy
		Application of theory to work
		Problem finding and solving
	Attitude at work	Learning desire
		Plasticity
		Understanding of professional ethics
	Career planning and confidence	Understanding and planning of individual career development
		Understanding of environment and development of industries
		Job search and self-promotion
Teacher knowledge transfer	Explicit knowledge	General overviews
		Specific requirements and data.
		Techniques.
		Progress and reports
		Project results
	Tacit knowledge	Teacher shares his/her job experience with me
		Teacher shares his/her expertise at the request of mine
		Teacher shares his/her ideas about jobs with me
		Teacher talks about his/her tips on jobs with me
Self-efficacy	Self-efficacy	I can remain calm when facing difficulties in my job because I can rely on my abilities
		When I am confronted with a problem in my learning tasks, I can usually find several solutions
		Whatever comes my way in my learning tasks, I can usually handle it
		My past experiences in my learning tasks have prepared me well for my occupational future
		I meet the goals that I set for myself in my learning tasks
		I feel prepared for most of the demands in my learning tasks
Deep approach to learning	Higher-order learning	Analyzed the basic elements of an idea, experience, or theory, such as examining a particular case or situation in depth and considering its components
		Synthesized and organized ideas, information, or experiences into new, more complex interpretations and relationships
		Made judgments about the value of information, arguments, or methods, such as examining how others gathered and interpreted data and assessing the soundness of their conclusions
		Applied theories or concepts to practical problems or in new situations
	Integrative learning	Worked on a paper or project that required integrating ideas or information from various sources
		Included diverse perspectives (different races, religions, genders, political beliefs, etc.) in class discussions or writing assignments
		Put together ideas or concepts from different courses when completing assignments or during class discussions
		Discussed ideas from your readings or classes with faculty members outside of class
		Discussed ideas from your readings or classes with others outside of class (students, family members, coworkers, etc.)
	Reflective learning	Examined the strengths and weaknesses of your own views on a topic or issue
		Tried to better understand someone else's views by imagining how an issue looks from his/her perspective

### Data Analysis Strategy

This study tested the hypotheses of the research framework and included paths *via* structural equation modeling. For higher-order constructs (teacher knowledge transfer, deep approach to learning, student employability), we reduced the number of parameters to be estimated following the partial aggregation method (Little et al., 2002). This procedure involves averaging the responses of subsets of items measuring a construct. In the measurement model, we first measured all dimensions and provided rigorous confirmatory factor analysis (CFA) report. Then, we averaged responses of each dimension to serve as indicators for these constructs in order to simplify the model and enhance the model fitting, because teacher knowledge transfer, deep approach to learning, and student employability were multidimensional constructs. Structural validity analysis was performed using IBM-AMOS statistical program, v. 23.0 for Windows. PLS-SEM (partial least squares structural equation modeling) was adopted to construct the structural model; specifically, verification of the structural model was performed using SmartPLS 3.0 (path analysis).

## Results

### Measurement Model

All scales used in this study were found to be reliable, with Cronbach α ranging from 0.83 to 0.96. Table 3 shows the reliability of each scale, the reliabilities in the instrument have been good, with a Cronbach α of >0.70. In order to gauge validity, this study employed CFA using AMOS 23.0 to verify the construct validity (both convergent and discriminant) of the scales. According to Hair et al.'s (2010) recommended validity criteria, CFA results show the standardized factor loading is higher than 0.7; average variance extracted (AVE) ranges between 0.539 and 0.729, and composite reliability ranges between 0.800 and 0.918. All three criteria for convergent validity were met, and correlation coefficients were all less than the square root of the AVE within one dimension, suggesting that each dimension in this study had good discriminant validity.

**Table 3:** Measurement properties.

		**1**	**2**	**3**	**4**	**5**	**6**	**7**	**8**	**9**	**10**
1. EK		***0.86/0.83***	0.724	0.440	0.489	0.437	0.369	0.376	0.346	0.351	0.271
2. TK		0.840	***0.88/0.88***	0.466	0.443	0.426	0.304	0.274	0.291	0.349	0.258
3. Self-efficacy		0.558	0.532	***0.82/0.81***	0.502	0.484	0.360	0.431	0.452	0.492	0.403
4. HOL		0.661	0.626	0.697	***0.88/0.86***	0.769	0.633	0.386	0.449	0.482	0.414
5. IL		0.566	0.550	0.628	0.805	***0.80/0.77***	0.667	0.427	0.437	0.476	0.420
6. RL		0.560	0.551	0.601	0.750	0.745	***0.92/0.92***	0.305	0.356	0.402	0.328
7. GAW		0.499	0.500	0.503	0.533	0.521	0.516	***0.72/0.72***	0.627	0.617	0.502
8. PAW		0.489	0.483	0.502	0.523	0.492	0.466	0.814	***0.84/0.80***	0.705	0.552
9. AW		0.545	0.556	0.561	0.576	0.547	0.532	0.757	0.747	***0.84/0.82***	0.654
10. PAC		0.474	0.478	0.558	0.537	0.547	0.492	0.656	0.640	0.744	***0.88/0.88***
Mean	Taiwan	3.889	3.849	3.754	3.687	3.587	3.688	3.536	3.642	3.605	3.557
	Malaysia	3.775	3.745	3.702	3.631	3.521	3.612	3.482	3.599	3.582	3.325
SD	Taiwan	0.657	0.679	0.624	0.650	0.649	0.686	0.640	0.701	0.704	0.726
	Malaysia	0.532	0.556	0.530	0.558	0.528	0.640	0.507	0.540	0.555	0.600
α	Taiwan	0.915	0.903	0.901	0.901	0.858	0.808	0.867	0.857	0.787	0.855
	Malaysia	0.888	0.901	0.894	0.884	0.827	0.828	0.802	0.807	0.759	0.822
AVE	Taiwan	0.746	0.775	0.669	0.770	0.639	839	0.525	0.700	0.702	0.775
	Malaysia	0.691	0.772	0.655	0.742	0.594	0.853	0.518	0.635	0.675	0.783
CR	Taiwan	0.936	0.932	0.924	0.931	0.898	0.912	0.897	0.903	0.876	0.912
	Malaysia	0.918	0.931	0.919	0.920	0.879	0.921	0.865	0.874	0.861	0.894

*The diagonal value is the square root value of AVE; left value belongs to Taiwanese sample and right value belongs to Malaysian sample. Correlation coefficients below the diagonal belong to the Taiwanese sample, and those above the diagonal belong to the Malaysian sample. AVE, average variance extracted; CR, composite reliability; EK, explicit knowledge; TK, tacit knowledge; HOL, higher-order learning; IL, integrative learning; RL, reflective learning; GAW, general ability for work; PAW, professional ability for work; AW, attitude at work; CPC, career planning and confidence. The diagonal bold and italic values are the square root values of AVE*.

### Inner Model Analysis

As for the assessment of the structural model, there is a suggestion by Hair et al. (2017) that, based on a resample of 5,000, the *R*^2^, β, and the corresponding *t* values are referred to by a bootstrapping algorithm. Not only these basic measures, but also the predictive relevance (*Q*^2^) and the effect sizes (*f*^2^) should be mentioned by researchers. Furthermore, it is available to make affirmation of the values of the variance inflation factor (VIF) prior to hypotheses testing. And the values of VIF are <5, which are changing from the scope of 1 and 2.032. Hence, no multicollinearity problems occur in the predictor latent variables (Hair et al., 2017).

**Figure 2:**
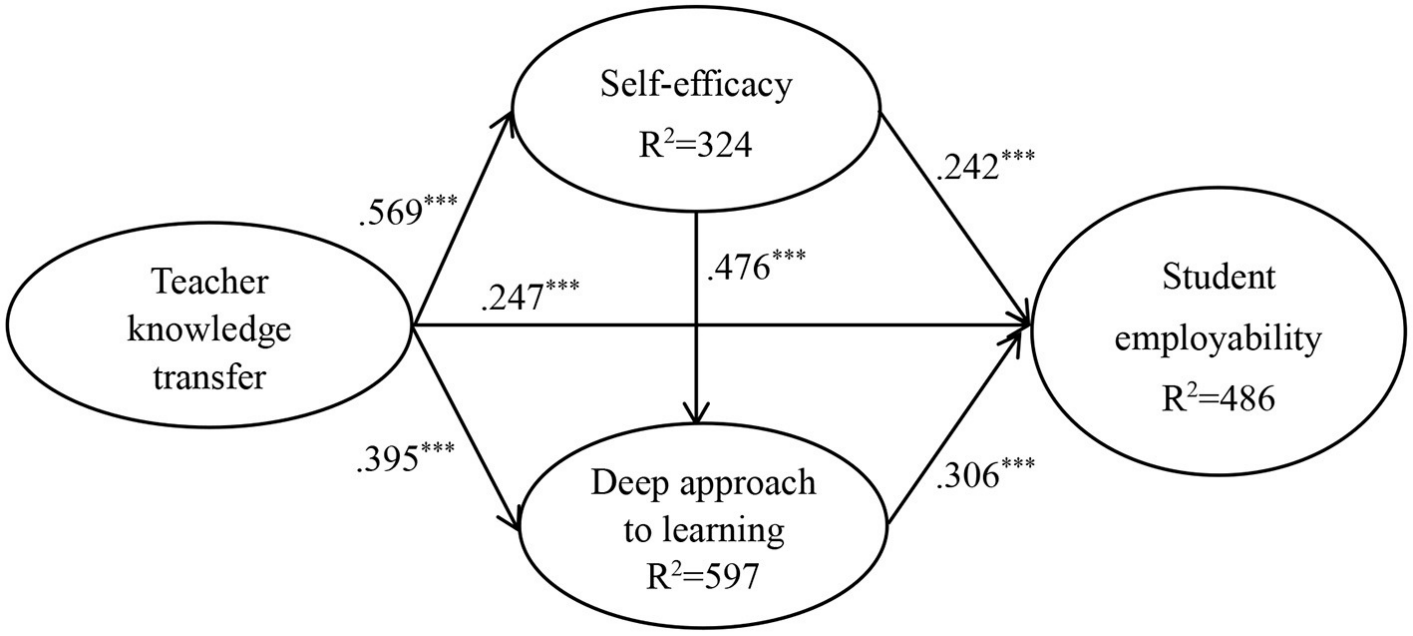
Structural model on Taiwanese student.

**Figure 3:**
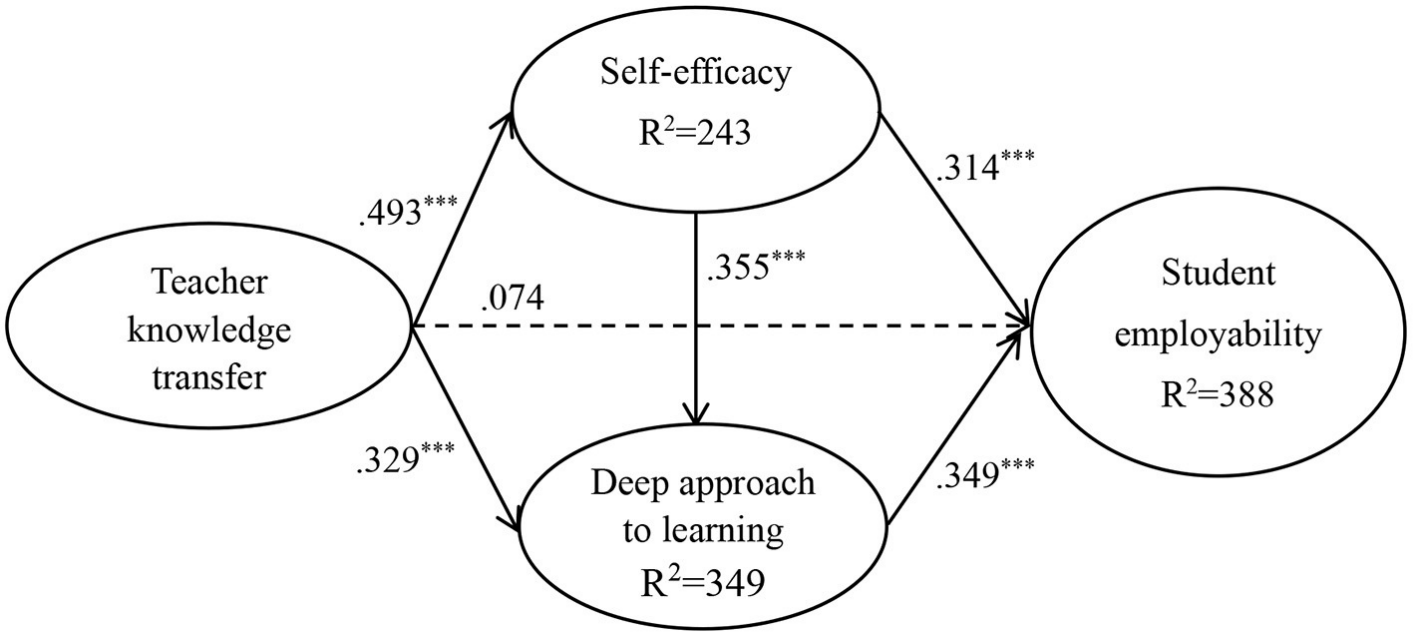
Structural model on Malaysian students.

Figures 2, 3 show the results of the hypothesized relationships and standardized coefficients in Taiwanese and Malaysian samples. The results showed that teacher knowledge transfer was positively and significantly related to student employability (β_Taiwan_ = 0.247, *f*^2^ = 0.064, *p* < 0.001; β_Malaysia_ = 0.074, *f*^2^ = 0.006, *p* > 0. 1) in Taiwanese rather than in Malaysian sample, partially supporting H1. Teacher knowledge transfer (β_Taiwan_ = 0.569, *f*^2^ = 0.480, *p* < 0.001; β_Malaysia_ = 0.493, *f*^2^ = 0.322, *p* < 0.001) was positively and significantly related to self-efficacy, supporting H2. Similarly, teacher knowledge transfer (β_Taiwan_ = 0.395, *f*^2^ = 0.262, *p* < 0.001; β_Malaysia_ = 0.329, *f*^2^ = 0.126, *p* < 0.001) was positively and significantly related to deep approach to learning, supporting H3.

In addition, self-efficacy (β_Taiwan_ = 0.242, *f*^2^ = 0.056, *p* < 0.001; β_Malaysia_ = 0.314, *f*^2^ = 0.106, *p* < 0.001) was positively and significantly related to student employability, supporting H4. Similarly, the path of self-efficacy → deep approach to learning (β_Taiwan_ = 0.476, *f*^2^ = 0.380, *p* < 0.001; β_Malaysia_ = 0.355, *f*^2^ = 0.146, *p* < 0.001) showed that the relations were positive and significant in Taiwanese and Malaysian samples, therefore supporting H5. Finally, the paths of deep approach to learning → student employability (β_Taiwan_ = 0.306, *f*^2^ = 0.074, *p* < 0.001; β_Malaysia_ = 0.349, *f*^2^ = 0.130, *p* < 0.001) showed that the relations were positive and significant in both samples, supporting H6. The Stone-Geisser *Q*^2^ values obtained through the blindfolding procedures for self-efficacy (*Q*^2^ = 0.186), EE (*Q*^2^ = 0.335), deep approach to learning (*Q*^2^ = 0.394), and student employability (*Q*^2^ = 0.326) were larger than zero, supporting that the model has predictive relevance (Hair et al., 2017).

### Multiple Group Analysis: Taiwan and Malaysia

It is confirmed that the measurement model is acceptable. However, in order to avoid overgeneralizing the data-driven patterns and theories, the study followed the suggestion of Hair et al. (2010) to divide the sample data into two groups based on regions (619 Taiwanese and 443 Malaysia students, respectively). Table 4 indicates the structural models' results and multiple group analysis (MGA) by using non-parametric methods including Henseler's MGA as recommended by Henseler et al. (2015). Despite the several differences in terms of significant path estimates between the groups, as indicated in Table 4, the multigroup permutation tests (final column on the right) show there are three significant differences between Taiwanese and Malaysian samples on all the paths. Specifically, in the structural model of Taiwanese students, all paths had significantly positive effects. However, comparing to Taiwanese students, in the structural model of Malaysia students, teacher knowledge transfer appeared to have no significant effects on student employability. This suggests that the Taiwanese students achieved greater student employability development from having well-established teacher knowledge transfer.

**Table 4:** Multigroup analysis result.

**Path**	**Path coefficients** **(confidence interval)**		**|β_Taiwan_ – β_Malaysia_|**	***p*-value** **Henseler's MGA**
	**β_Taiwan_** **(2.5–97.5%)**	**β_Malaysia_** **(2.5–97.5%)**		
H1: Teacher knowledge transfer → student employability	0.247 (0.170–0.317)	0.074 (−0.041–0.182)	0.173	0.005
H2: Teacher knowledge transfer → self-efficacy	0.569 (0.501–0.630)	0.493 (0.418–0.568)	0.076	0.071
H3: Teacher knowledge transfer → deep approach to learning	0.395 (0.319–0.469)	0.329 (0.214–0.422)	0.067	0.153
H4: Self-efficacy → student employability	0.242 (0.154–0.331)	0.314 (0.205–0.412)	0.072	0.856
H5: Self-efficacy → deep approach to learning	0.476 (0.391–0.555)	0.355 (0.241–0.448)	0.121	0.035
H6: Deep approach to learning → student employability	0.306 (0.204–0.401)	0.349 (0.230–0.459)	0.043	0.714

## Conclusions

### Discussions

In previous discussions of student employability, many studies have focused on the antecedents of students' individual behavior patterns and psychological cognition (Ahmed et al., 2015; Cacciolatti et al., 2017; Blázquez et al., 2018). This study takes Taiwanese and Malaysian students as research samples to verify whether teacher knowledge transfer positively affects student employability, assuming that teacher knowledge transfer has a direct effect on self-efficacy, deep approach to learning, and student employability in the SCCT model. This study will fill the theoretical gap in the application of Western theories under the Eastern context and increase the generalization of the theory. Based on our research findings, this study provides the following contributions. First, the results support that the positive concept was interpreted through teacher knowledge transfer. Through TK and EK gaining, students can achieve greater learning engagement and can adopt effective learning methods. Second, most previous studies on the SCCT explored the importance of environmental factors, but only a few studies provided essential contributions in the comparison of cross-country and cross-culture (Presti et al., 2018). This study aims to fill the theoretical gap and enrich the theoretical foundation of the SCCT. Third, scholars have claimed that repeated verification of the theory in different situations helps to enrich the development of the theory. However, as the theory matures over time, it should begin to focus on the compatibility and combination of theories. This study aims to combine knowledge management and the SCCT into higher education to explore students learning, which will improve the compatibility of the SCCT with other theories.

The results indicate that the teacher knowledge transfer in the Taiwanese sample was positively related to student employability, whereas there was no significant effect in Malaysian students. These results correspond with Cupani et al. (2010) and Lent et al. (2016); on the basis of the SCCT, they believe that the learning environment differences between countries or cultures influence students' learning status and learning activities, causing knowledge and skills-gaining to differ. Our findings are consistent with those of previous studies (Brown et al., 2011; Duffy et al., 2014; Burga et al., 2020), which suggests that the SCCT model is relevant across numerous areas. Besides, there may be an insignificant correlation between teacher knowledge transfer and student employability in Malaysian students; the reasons may indicate that differences in knowledge transfer and learning process influence the extent to which students absorb sufficient knowledge to foster suitable employability. Conforming to the results of Burga et al. (2020) and Lent et al. (2007, 2012), this conclusion indicates the significant position of self-efficacy and deep approach to learning in the SCCT model, as well as the impact on the development of student employability that must be noticed.

Moreover, the results indicate positive correlations among teacher knowledge transfer, self-efficacy, and deep approach to learning for both Taiwanese and Malaysian students. It is worth noting that the role of teachers in knowledge transfer implies that students with more TK and EK from teachers are willing to become involved in the learning environment and actively participate in learning activities (Steins and Behravan, 2017), thus obtaining the ability and confidence to achieve course tasks, such as the development of systematic/integrative thinking and problem-solving skills. This finding is consistent with a number of previous studies (Pike et al., 2012; Bocanegra et al., 2016), supporting the relationship among teacher knowledge transfer, self-efficacy, and deep approach to learning.

Our results also found that self-efficacy was positively and significantly related to deep approach to learning and student employability for both Taiwanese and Malaysian students. It is notable that this result goes in line with the arguments of previous SCCT studies (Lent and Brown, 2013), in which students adopt self-efficacy in a given domain arising from learning experiences for confidence measurement while doing subsequent learning involvement and performance (Komarraju and Nadler, 2013; Burga et al., 2020). However, unlike Dacre Pool and Qualter's (2013) research, the present study took junior and senior students as the subjects, rather than exploring the relationship between the self-efficacy and employability of serving staff. Therefore, the results overcome the lack of student samples in the SCCT satisfaction model and employability theory (Burga et al., 2020). Being psychosocial, employability can be accounted for *via* the social cognitive variables of self-efficacy, which conforms to research by Bandura (1995). Moreover, to promote task organization, management, and execution, individuals turn to strong self-efficacy establishment (Burga et al., 2020), through which a socialized model of learning and SE promotion has been developed. The positive and direct impact of deep approach to learning on student employability development is exposed by the findings. There is a high correlation between deep approach to learning utilization and employability, and deep approach to learning utilization leads to the facilitation of positive behaviors and capacities. This is possibly explained by the fact that deep approach to learning makes students construct their own knowledge and abilities (Campbell and Cabrera, 2014; Dolmans et al., 2016; Varunki et al., 2017), so knowledge acquisition and integration can be meaningfully linked with useful knowledge. Although deep approach to learning can significantly affect the acquisition of professional knowledge, there has been no accordant result for student achievement (Dolmans et al., 2016). The identification of the diversified needs in different areas of expertise for deep approach to learning may be created by differences in the research samples.

Finally, our results showed that there was a significant difference between Taiwan and Malaysia in the same SCCT model. These findings are quite consistent with those of Lent et al. (2016) and Sheu et al. (2014), who verified the well-being model cross-sectionally in different samples of college students. Unlike previous studies using the SCCT model (Brown et al., 2011; Duffy et al., 2014; Burga et al., 2020), this study verifies learning process and employability training of students from two countries with similar culture. The SCCT model emphasizes the importance of environmental conditions, but previous literature rarely discussed and described the multicultural context. This study discusses this context and makes comparison in the model, which provides more diversified discussions and insights for SCCT and enriches SCCT's theoretical foundation. Because of cross-cultural differences, Taiwanese students are aware of a high degree of personal obligation in the face of teacher knowledge transfer and employment market information collection. Therefore, it is easy to detect the role that they should play as having a positive impact on the participation of various learning activities. Moreover, different from the study of Sheu et al. (2014), this study compared samples of different regions in the same model, that is, Taiwanese and Singaporean college students, reporting good overall model-data fit in both samples (Taiwan and Malaysia) and verifying direct and indirect effects of self-efficacy generated in the SCCT on student employability. However, differing from the studies of Lent et al. (2016) and Sheu et al. (2014), this study also considered the combination of several theoretical foundations and enriches the theoretical model of the SCCT in employability based on the region analysis.

### Implications

In summary, according to our findings, this study suggests some important practical implications for improving the quality of higher education. First, in this study, teacher knowledge transfer was perceived as equally important and predictive of students' own perceived levels of self-efficacy, deep approach to learning, and student employability. Thus, in the face of global employment challenges all over the world, institutional administrations should encourage teachers to actively form close connections with students, build communication platforms using technological media and information technology tools, and provide schoolwork or psychological support in real time.

Second, a high degree of learning engagement may affect students to adopt effective learning modes, such as deep approach to learning. Thus, administrations must be focused on to improve learning engagement. On this basis, this study suggests that institutional administrations should create an effective learning environment in cooperation with teachers. To create a learning environment that allows for students to adopt deep approach to learning, in addition to improving through the use of technological media, HEIs may facilitate greater teacher collaboration, enabling easier sharing of teachers' TK and EK and other information.

Third, in light of the structural patterns of the two regions, student employability and self-efficacy derived from the teacher knowledge transfer of the Taiwanese sample are superior to the Malaysian sample. This implies that students in Taiwan have more concerns about knowledge exploration and exploitation; they emphasize specifically how to reuse knowledge in different situations, how to utilize knowledge in problem-solving, and how to leverage knowledge in RL. Thus, this study suggests that Malaysian institutional administrations should enable students to develop these capabilities through the transfer of particular knowledge to cope with problems resulting from course tasks because what students need is not courses but the method to solve existing problems.

### Research Limitations

The literature concerning with cross-cultural study, SCCT, and student employability benefits from the study findings, but some limitations still exist. First, in the field of psychology and education, the SCCT has been emphasized; however, there are a few studies in which the relationship between teacher knowledge transfer and student employability has been taken into account. Despite the construction of teacher knowledge transfer in relation to the SCCT in this study, other cognition theories have been used in learning in region-specific students, such as theories of social exchange (Fan et al., 2019), knowledge conversion (Astorga-Vargas et al., 2017), and self-regulated learning (Broadbent, 2017). To identify related dimensions of psychology that have an effect on student employability, it is suggested that multiple theoretical models be employed in subsequent studies. Second, details from students regarding teacher knowledge transfer require self-reporting, and this may result in sampling and nonresponse errors when students provide statements of their psychological status. If assessments of the actual psychological status of students are available, it may lead to a better understanding of the connection between knowledge transfer and the employability of students. Besides, in this study, despite that there is no difference in all the variables between social and natural disciplines, previous studies have indicated that different disciplines and students' genders result in different research outcomes; thus, it is necessary to incorporate disciplines and genders into issues related to student learning. As the study aims to strengthen the degree of theoretical generalization, the influence that gender and discipline bring to the research model is not discussed in the research findings. As a consequence, it is suggested that subsequent researchers can add the variable of students' background for comparative analysis to provide more valuable insights and enrich theoretical connotations. In addition, future researchers should conduct interviews and observations of learning status to obtain support for the study findings and conclusions. Third, in the study, we have used a sample of only 20 universities with a total of 1,062 valid copies of questionnaire. We have taken Taiwanese and Malaysian students as objects for study. Studies in other countries and improvements in the quantity expansion of samples and research representativeness are necessary for other insights into talent education enhancement. Furthermore, knowledge transfer theory contains the interaction pattern between knowledge providers and knowledge receivers. In this study, only students were sampled, and the effect brought by knowledge transfer and the influence on subsequent variables have been explored from the aspect of students, but the knowledge providers (i.e., teachers) have not been surveyed. In this regard, the study suggests that subsequent researchers can add teachers in the questionnaire to conduct cross-level hierarchical model analysis, so as to know the actual interactive conditions and situations between teachers and students and then enrich the significance of practice. Finally, statements and hypotheses based on the comparative analysis of Taiwan and Malaysia are not proposed, because this study puts emphasis on the cross-cultural comparison. Thus, we suggest researchers to include literature on cross-regional comparison based on the research findings of this study and propose verifiable hypotheses for theoretical development.

## Data Availability Statement

The raw data supporting the conclusions of this article will be made available by the authors, without undue reservation.

## Ethics Statement

The studies involving human participants were reviewed and approved by Ethics Committee in Taipei of University. The patients/participants provided their written informed consent to participate in this study.

## Author Contributions

This study was a joint work of all authors. MY-P contributed to the ideas of research, collection of data, and empirical analysis. W-XZ contributed to writing, data analysis, and design of research methods and tables. FL participated in developing a research design, proof writing, and interpreting the analysis. All authors contributed to the literature review and conclusions.

## Conflict of Interest

The authors declare that the research was conducted in the absence of any commercial or financial relationships that could be construed as a potential conflict of interest.
